# Ethics training competencies and leadership enable responsible AI in hospitality and tourism

**DOI:** 10.1016/j.isci.2026.115134

**Published:** 2026-02-27

**Authors:** Muhammad Arif, Liyang Zhao, Xiaoyan Zou, Keshun Li, Zhangting Chen, Ye Ai, Mengyao Tian

**Affiliations:** 1 School of Tourism Ecology and Environment, Guilin Tourism University, Guilin 541006, China; 2 Guangxi Cultural Tourism and Wellness Integrated Development Center, Guilin Tourism University, Guilin 541006, China

**Keywords:** Business, Information science

## Abstract

Artificial intelligence (AI) is reshaping the service industries, increasing demand for fair, transparent, and ethical systems. This study examined how AI ethics training, employee competencies, organizational governance maturity, and leadership commitment jointly supported responsible AI performance. Survey data from employees in hospitality and tourism organizations were analyzed using structural equation modeling. The findings indicated that ethics training was associated with stronger responsible AI performance through two complementary mechanisms: the development of employee competencies and the institutionalization of governance processes. Leadership commitment further strengthened the relationship between governance maturity and performance, highlighting the role of organizational values in ethical technology use. Together, these results indicated that responsible AI emerged from the alignment of learning practices, institutional structures, and leadership priorities. The study provides practical guidance for service organizations seeking to embed ethical principles into AI use. It contributes to broader discussions about responsible digital transformation and sustainable service innovation.

## Highlights

• AI ethics training improved responsible AI performance in service firms
• Competencies and governance maturity transmitted training effects to performance
• Leadership AI ethics commitment strengthened maturity links to performance
• Responsible AI emerged from aligned learning practices across governance and leadership

## Graphical abstract

**Figure:**
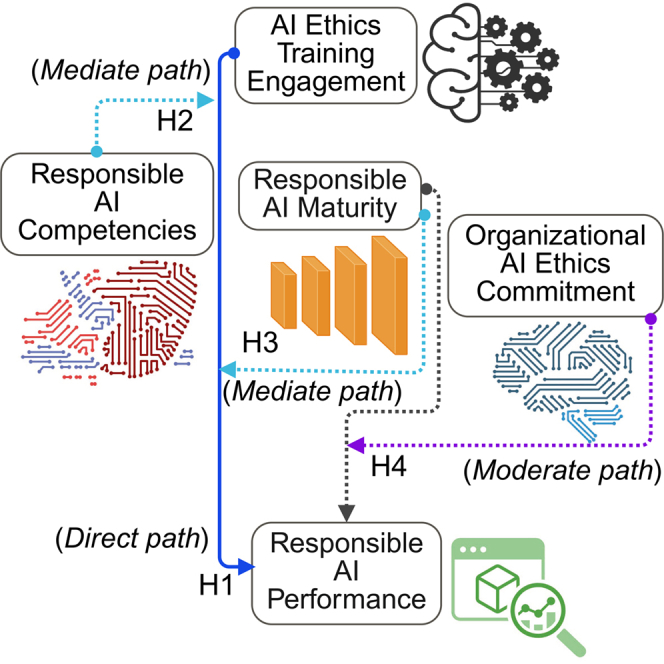

## Introduction

Sustainability is widely recognized as one of the defining challenges of the twenty-first century.^1^^,^^2^^,^^3^ It has direct implications for human well-being, social resilience, and long-term economic competitiveness.^4^^,^^5^ Environmental degradation alone is projected to contribute to more than 250,000 deaths annually by 2050, which highlights the severity of the problem.^6^^,^^7^ As a result, governments, industries, and researchers have increasingly turned their attention to climate action, resource efficiency, and responsible technological innovation.^8^^,^^9^^,^^10^ Within service-based economies, sustainability has shifted from a desirable aspiration to a strategic necessity, because responsible practices enhance competitiveness, strengthen reputation, and build trust among stakeholders.^11^ Digital technologies have played an important role in this transition. Tools such as digital supply chain monitoring, eco-certification platforms, and analytics-driven reporting systems have enabled organizations to optimize resource use and improve employee engagement with sustainability programs.^12^^,^^13^ Despite these benefits, scholars increasingly argue that digital transformation alone cannot guarantee ethical, transparent, or socially responsible outcomes.^3^^,^^14^ Emerging technologies, especially artificial intelligence (AI), can introduce new vulnerabilities and intensify existing inequalities when not guided by strong governance systems. These concerns signal a shift from a technology adoption perspective toward frameworks that prioritize ethics, accountability, and organizational responsibility.

AI is rapidly reshaping the service industries and altering how firms engage with customers, manage employees, and coordinate complex service processes.^15^ In hospitality and tourism, AI supports demand forecasting, personalized service delivery, workforce scheduling, and sustainability reporting.^16^^,^^17^ However, these opportunities coexist with significant ethical challenges. Issues such as algorithmic bias, opacity, privacy violations, and accountability failures can create risks for individuals and for a broader society.^18^ When organizations lack effective governance frameworks, AI systems may unintentionally amplify discrimination or compromise fundamental trust in service relationships.^19^ These risks are particularly relevant in the service industries because they rely heavily on interpersonal interactions. Unlike manufacturing, where automation primarily affects routine tasks, AI in services influences the quality of human relationships, employee well-being, and customer experience. Examples include biased AI-driven recruitment, opaque recommendation algorithms within tourism platforms, and intrusive monitoring tools used in hospitality. Such cases emphasize the growing need for responsible AI governance, which refers to institutional systems that integrate ethical reasoning, human oversight, and accountability throughout the AI life cycle.^2^

Responsible AI, also known as ethical AI, emphasizes principles such as fairness, accountability, transparency, and sustainability.^20^^,^^21^ Global initiatives that include the European Union’s Ethics Guidelines for Trustworthy AI and UNESCO’s Recommendation on AI Ethics reflect these priorities. However, organizations often face considerable challenges when attempting to translate these high-level principles into practice.^22^ Successful implementation requires capacity building at both the individual and organizational levels. Human resource management (HRM) has consequently become an important domain for operationalizing responsible AI. AI ethics training helps employees understand algorithmic risks, apply fairness concepts, and make informed decisions in AI-supported environments.^23^ Such training encourages workers to view AI as a socio-technical system shaped by human values and institutional norms, rather than as a purely technical innovation.^1^^,^^24^ The broader sustainability and digital transformation literature consistently identifies training as a central driver of innovation and long-term organizational performance. In the context of green HRM, employee training has been shown to foster pro-environmental behavior, strengthen competencies, and promote organizational learning.^25^^,^^26^ Applying these insights to AI, ethics training is expected to promote responsible AI competencies, which include the skills, knowledge, and awareness necessary to identify risks, interpret AI outcomes, and apply ethical principles in decision-making.^27^ These competencies shape how training influences organizational outcomes and determine whether ethical considerations are applied consistently in practice.

However, organizational processes also play a critical role. Ethical AI use cannot depend solely on individual learning. It requires formal governance systems that include risk assessment procedures, oversight structures, performance accountability mechanisms, and ongoing evaluation. An investigation is needed to conceptualize these organizational systems within the framework of responsible AI maturity. This construct can be informed by maturity models in sustainability research, which describe how organizations progress from minimal compliance toward proactive integration of ethical practices.^7^^,^^9^^,^^28^ As AI maturity increases, organizations embed responsible AI principles more deeply into their strategy, structure, and daily routines. Leadership commitment is another major factor influencing responsible AI performance.^29^ Leadership commitment captures the extent to which senior managers actively support ethical AI use through communication, policy development, and resource allocation. Prior sustainability research demonstrated that leadership engagement strengthens the link between organizational maturity and performance.^14^ Similar patterns are expected in responsible AI governance. Leaders who prioritize ethical AI create an environment in which employees are more likely to apply ethical principles, and governance frameworks are more likely to be implemented effectively.^30^ In contrast, low levels of commitment can undermine training effectiveness and weaken ethical practices institutionalization.

Although responsible AI has gained prominence in policy debates and scholarly discussions, empirical work in the service industries remains limited. Much of the existing literature focuses on technology firms, manufacturing contexts, or consumer-facing AI applications.^31^^,^^32^^,^^33^ Few studies examine how human resources practices influence responsible AI use or how competencies, governance maturity, and leadership values interact within service organizations. These research gaps are critical in rapidly digitalizing countries such as China, where AI adoption is closely linked to national innovation and sustainability objectives.^17^^,^^34^ Hospitality and tourism organizations are especially relevant because they operate in data-intensive environments and rely heavily on relational values and reputations. Hotels, travel platforms, and tour operators increasingly use AI for recruitment, personalization, and sustainability management in ways that significantly influence employees and customers.^35^ Yet empirical evidence on how such organizations develop responsible AI practices remains scarce.

This study aims to address these gaps by developing and testing an integrated framework that links ethics training, responsible AI competencies, responsible AI maturity, and leadership commitment to responsible AI performance. The framework draws on resource-based and institutional perspectives to explain how training and governance structures support capability building and organizational legitimacy. The study contributes to the literature in three ways. First, it extends research into HRM and responsible innovation by conceptualizing ethics training as a strategic mechanism that supports responsible digital transformation. Second, it introduces responsible AI competency and maturity as mediating mechanisms that explain how training influences organizational outcomes. Third, it examines the moderating effect of leadership commitment to identify the conditions under which maturity is most strongly associated with responsible AI performance. The study uses partial least squares structural equation modeling (PLS-SEM) to empirically evaluate the proposed relationships. By focusing on hospitality and tourism enterprises within the broader global service ecosystem, the study brings empirical evidence to debates about responsible AI governance. It offers insightful advice for managers, human resource professionals, and policymakers.

### Literature review

**Figure 1:**
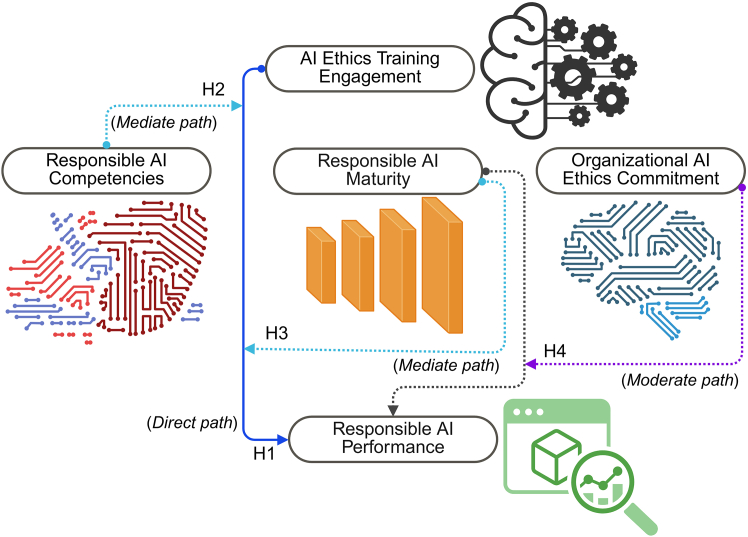
Conceptual framework illustrating the influence of AI ethics training on responsible AI performance in service firms Created at https://BioRender.com.

Responsible AI is increasingly conceptualized as an organizational capability that depends on the integration of ethical principles, governance structures, and human-centered decision-making. Although rapid technological advances have enabled widespread adoption of AI across service operations, prior research emphasized that responsible and transparent deployment relies not only on technical design but also on individual competencies and institutional maturity.^20^^,^^21^ This distinction is particularly salient in the service industries, where customer interaction and employee judgment are central to value creation. In such contexts, failures related to fairness, transparency, or accountability can directly undermine service quality, trust, and organizational legitimacy.^18^^,^^19^ This literature review outlines the theoretical foundations of responsible AI performance and synthesizes prior research across four interrelated constructs: AI ethics training, responsible AI competencies, responsible AI maturity, and organizational commitment to AI ethics. Together, these constructs form the basis of the conceptual framework presented in Figure 1. The model proposes that ethics training contributes to responsible AI performance directly and indirectly through competencies and maturity. In addition, organizational commitment strengthens the effect of maturity on performance.

### Responsible AI performance

Responsible AI performance refers to the extent to which organizations deploy AI systems in ways that are fair, transparent, accountable, and aligned with ethical and societal values. This performance dimension encompasses practices that reduce algorithmic bias, ensure meaningful human oversight, protect data privacy, and generate outcomes consistent with stakeholder expectations.^18^^,^^36^ In service ecosystems such as hospitality and tourism, responsible AI performance is particularly critical because technology-mediated decisions often affect both employees and customers simultaneously, amplifying reputational and relational risks.^17^^,^^29^ Although global policy frameworks such as the Ethics Guidelines for Trustworthy AI and UNESCO’s Recommendation on AI Ethics articulate high-level principles, empirical evidence explaining how organizations operationalize these principles remains limited. Existing studies tend to emphasize technical attributes or regulatory compliance, rather than the organizational and human mechanisms through which ethical intentions translate into observable performance outcomes.^35^ Accordingly, responsible AI performance is treated in this study as the focal outcome variable. This illustrates the extent to which AI-supported service processes incorporate ethical standards.

### AI ethics training

Training has long been recognized as a core mechanism for shaping employee knowledge, ethical reasoning, and technology-related behavior. In sustainability and green HRM research, training initiatives enhance environmental competencies, promote responsible conduct, and support organizational learning.^25^^,^^37^ Extending this logic, AI ethics training equips employees with an understanding of algorithmic processes, ethical risks, fairness concerns, and appropriate decision-making practices when interacting with AI systems.^23^ Beyond technical awareness, ethics training frames AI as a socio-technical system shaped by human values, organizational culture, and data practices, rather than as a neutral or autonomous technology.^1^^,^^24^ Through scenario-based learning, simulations, and reflective exercises, the training encourages employees to recognize situations where algorithmic outputs require scrutiny or human intervention. Consequently, the literature positions AI ethics training as a foundational organizational ,mechanism that shapes employee behavior and establishes shared expectations regarding ethical AI use. While training alone is insufficient to ensure ethical outcomes, it provides the conditions under which employees can apply ethical principles in AI-supported work environments. Building on sustainability-oriented HRM research, this study proposes the following hypothesis.

H1. AI ethics training engagement has a significant positive effect on responsible AI performance in service firms.

### Responsible AI competencies

Responsible AI competencies refer to the knowledge, skills, and ethical awareness that enable employees to interact with AI systems responsibly. These competencies include the ability to identify potential biases, critically interpret algorithmic outputs, recognize transparency limitations, and apply ethical judgment in service-related decisions.^27^ In HRM research, competencies function as key mechanisms through which training influences organizational performance, innovation, and responsible behavior.^9^^,^^16^ Within service ecosystems, employees with well-developed responsible AI competencies are better positioned to evaluate algorithmic recommendations, escalate ethical concerns, and maintain accountability when AI is applied to recruitment, customer service, or operational management.^15^ Because responsible AI depends on informed human oversight rather than automation alone, competencies represent a critical pathway linking ethics training to performance outcomes. This hypothesis reflects the expectation that training enhances employee competencies, which in turn support responsible AI use in practice.

H2. Responsible AI competencies mediate the relationship between AI ethics training engagement and responsible AI performance.

### Responsible AI maturity

Responsible AI maturity captures the extent to which ethical principles and governance mechanisms are systematically embedded within organizational processes related to AI use. Maturity encompasses formal structures such as risk assessment procedures, algorithmic auditing practices, internal communication protocols, and ethical review mechanisms that support accountability and oversight.^28^ Maturity models in sustainability research describe organizational progression from basic compliance toward structured integration and proactive responsibility.^7^^,^^9^ Applied to AI governance, maturity reflects institutional capacity to monitor algorithmic behavior, mitigate risks, and ensure the consistent application of ethical standards. Training contributes to maturity by building awareness and readiness for governance practices, while organizations with higher maturity are better equipped to manage AI responsibly across functions and service processes.^38^^,^^39^ This hypothesis aligns with institutional theory, which emphasizes the role of structured governance systems in building legitimacy and accountability.

H3. Responsible AI maturity positively mediates the relationship between AI ethics training engagement and responsible AI performance.

### Organizational AI ethics commitment

Organizational commitment to AI ethics refers to the extent to which senior leaders actively prioritize ethical AI use through strategic communication, resource allocation, policy development, and cultural reinforcement.^29^ Leadership plays a central role in determining whether governance structures are implemented effectively and whether employees feel supported when applying ethical principles in their work.^30^ Research in sustainability and governance consistently shows that leadership commitment strengthens the relationship between organizational maturity and performance by clarifying priorities and reinforcing values.^14^ Similar mechanisms are expected in responsible AI governance. When leadership commitment is strong, governance systems are more likely to translate into meaningful ethical outcomes. When commitment is weak, even well-designed structures may fail to influence practice. This hypothesis captures the moderating role of leadership commitment within the conceptual framework presented in Figure 1.

H4. Organizational AI ethics commitment positively moderates the relationship between responsible AI maturity and responsible AI performance.

## Results

### Participant demographics

The demographic characteristics of the respondents provide useful insight into the composition of the workforce engaged in AI ethics training in Chinese hospitality and tourism enterprises. Female respondents accounted for 52.330% of the sample and male respondents for 47.670%, reflecting women’s strong participation in service sector roles and their growing representation in management and sustainability-related positions. A majority of participants were single at 58.065%, and the most represented age group was 18–30 years at 42.294%. Employees aged 31 to 40 accounted for 26.165%, those aged 41 to 50 represented 23.297%, and 8.244% were 51–60 years old. The educational profile indicated high academic attainment. More than half of respondents held bachelor’s degrees at 51.971%, while 41.577% possessed master’s degrees. This suggests a workforce with substantial intellectual readiness for advanced digital training and ethical technology integration. Professional experience also showed a balanced distribution, with 43.011% having 1 to 8 years of work experience and 37.634% having 9 to 16 years of experience. Only a small proportion exceeded 17 years. These demographics demonstrate that the sample represents a predominantly young and digitally competent workforce that is well positioned to engage with emerging AI governance practices. Demographic details are provided in Table 1.

**Table 1:** The demographic characteristics of respondents (*n* = 279) provide context for attitudes toward AI and green service innovation

Variable	Category	Number (N)	Percentage (%)	*p* value
Gender	Male	133	47.670	∗∗∗
	Female	146	52.330	
Marital status	Single	162	58.065	∗∗∗
	Married	117	41.935	
Age (years)	18–30	118	42.294	∗∗∗
	31–40	73	26.165	
	41–50	65	23.297	
	51–60	23	8.244	
Education	Senior school	18	6.452	∗∗∗
	Graduation	145	51.971	
	Post graduation	116	41.577	
Work experience (years)	1-8 years	120	43.011	∗∗∗
	9-16 years	105	37.634	
	17-24 years	35	12.545	

Note: The Kruskal-Wallis test indicates significant differences, with ∗∗∗*p* < 0.001.

### Construct reliability and convergent validity

Reliability and validity tests confirmed that the measurement model was statistically sound. As shown in Table 2, all constructs achieved Cronbach’s α values above 0.90, far surpassing the minimum threshold of 0.70, indicating very strong internal consistency. The Rho_A values were similarly high, with most exceeding 0.89, confirming stability across all constructs. Although AI ethics training engagement recorded a slightly lower value (0.892), it remained well above the acceptable level. Composite reliability values for all constructs ranged from 0.911 to 0.943, providing further evidence of consistency across items. Convergent validity was demonstrated through AVE. Organizational AI ethics commitment (0.639) and responsible AI performance (0.659) were both around the ideal 0.70 benchmark, indicating that their items strongly represented latent constructs. Responsible AI competences, training engagement, and maturity also met the minimum AVE criteria of 0.50, confirming their satisfactory validity. These results demonstrate that the measurement model was both reliable and valid, providing a robust foundation for subsequent structural analyses. While AVE scores for a few constructs were relatively lower than others, they remained within acceptable ranges. This fact implies that future studies can refine measurement precision, but the current instrument is suitable for empirical testing.

**Table 2:** Testing the validity and reliability of measurement items through confirmatory factor analysis

Code	Construct (Items)	Loading	*t* value
OAEC	Organizational AI ethics commitment (α = 0.884, CR = 0.911, AVE = 0.639)		
OAEC1	Leadership actively promotes responsible AI values within the organization.	0.827	∗
OAEC2	The organization has clear policies supporting AI ethics and accountability.	0.792	∗
OAEC3	Resources are allocated to ensure compliance with AI ethics standards.	0.839	∗
OAEC4	Management communicates the importance of AI ethics to employees.	0.778	∗
OAEC5	The organization’s long-term strategy prioritizes responsible AI adoption.	0.806	∗
OAEC6	Our firm regularly collaborates with stakeholders to advance responsible AI practices.	0.783	∗
RAIP	Responsible AI performance (α = 0.924, CR = 0.943, AVE = 0.659)		
RAIP1	Our AI systems comply with relevant laws and ethical standards.	0.84	∗
RAIP2	The organization actively reduces algorithmic bias in its AI solutions.	0.819	∗
RAIP3	Our AI-driven processes demonstrate transparency and accountability.	0.857	∗
RAIP4	Stakeholders trust the fairness of our AI systems.	0.792	∗
RAIP5	The adoption of AI has improved both organizational performance and ethical outcomes.	0.835	∗
RAIP6	Our organization has been recognized for responsible AI practices.	0.802	∗
RAIC	Responsible AI competencies (α = 0.914, CR = 0.936, AVE = 0.587)		
RAIC1	Employees are skilled at identifying potential bias in AI systems.	0.787	∗
RAIC2	Staff understand the importance of AI transparency and explainability.	0.801	∗
RAIC3	Employees can apply ethical AI guidelines in their daily work.	0.768	∗
RAIC4	Staff are capable of balancing innovation with ethical considerations.	0.815	∗
RAIC5	Our organization supports employees in developing skills for AI governance.	0.774	∗
RAIC6	Employees are confident in addressing ethical dilemmas arising from AI use.	0.793	∗
AETE	AI ethics training engagement (α = 0.891, CR = 0.923, AVE = 0.617)		
AETE1	Our organization provides regular training on ethical AI practices.	0.826	∗
AETE2	Employees have access to online platforms and tools for AI ethics learning.	0.795	∗
AETE3	Training programs include case studies on fairness, transparency, and accountability in AI use.	0.838	∗
AETE4	AI ethics training is tailored to fit our organization’s industry context.	0.779	∗
AETE5	I actively engage in organizational programs focused on responsible AI.	0.871	∗
AETE6	The organization encourages continuous learning on AI ethics issues.	0.786	∗
RAIM	Responsible AI maturity (α = 0.902, CR = 0.926, AVE = 0.574)		
RAIM1	Our organization conducts regular audits of AI systems for ethical compliance.	0.792	∗
RAIM2	Responsible AI principles are integrated into our governance framework.	0.779	∗
RAIM3	AI risk assessments are carried out before deploying new systems.	0.803	∗
RAIM4	The organization actively monitors and updates AI systems to ensure fairness and transparency.	0.768	∗
RAIM5	Ethical considerations are part of AI project planning and implementation.	0.784	∗
RAIM6	We are proactive in aligning AI systems with emerging regulations and global ethical standards.	0.743	∗
RAIM7	Our organization’s approach to AI ethics has evolved from compliance-driven to innovation-driven.	0.771	∗

Note: The table uses the following abbreviations: Cronbach’s alpha (α), composite reliability (CR), factor loading (λ), and average variance extracted (AVE). A *t* value is considered significant at ∗*p* < 0.05, corresponding to a value greater than 1.

### Evaluation of the measurement model

Multicollinearity tests (Table 3), conducted using VIFs, showed that all values were below the threshold of 5.0, with almost all under the conservative limit of 3.3. This demonstrated that the constructs were not compromised by redundancy, even when multiple items measured overlapping aspects of responsible AI.

**Table 3:** Variance inflation factor (VIF) assessment for measurement and structural model

Construct	Items/Path	VIF
Organizational AI Ethics Commitment (OAEC)	OAEC1	2.104
	OAEC2	2.345
	OAEC3	1.899
	OAEC4	2.208
	OAEC5	2.054
	OAEC6	1.763
Responsible AI Performance (RAIP)	RAIP1	2.457
	RAIP2	2.308
	RAIP3	2.112
	RAIP4	1.951
	RAIP5	2.143
	RAIP6	1.870
Responsible AI Competencies (RAIC)	RAIC1	2.145
	RAIC2	2.210
	RAIC3	2.114
	RAIC4	2.274
	RAIC5	2.156
	RAIC6	2.191
AI Ethics Training Engagement (AETE)	AETE1	2.134
	AETE2	2.298
	AETE3	2.177
	AETE4	2.201
	AETE5	2.031
	AETE6	2.842
Responsible AI Maturity (RAIM)	RAIM1	2.267
	RAIM2	2.334
	RAIM3	2.196
	RAIM4	2.362
	RAIM5	2.188
	RAIM6	2.097
	RAIM7	2.145
Structural model paths	AETE → RAIC	1.851
	AETE → RAIM	2.055
	RAIC → RAIM	2.279
	AETE → RAIP	2.507
	RAIC → RAIP	2.225
	RAIM → RAIP	2.353
	OAEC → RAIM → RAIP	2.108

Note: All VIF values ranged between 1.763 and 2.842, well below the recommended cut-off of 3.300. Table 2 explains items abbreviations.

Discriminant validity was assessed through both the Fornell-Larcker criterion and the heterotrait-monotrait ratio. Results confirmed that each construct was more strongly associated with its indicators than with the others. For instance, responsible AI performance correlated strongly with responsible AI maturity (r = 0.672), showing that firms with higher levels of maturity achieved stronger ethical AI outcomes (Table 4). Training engagement was positively correlated with both competencies (r = 0.609) and maturity (r = 0.571), suggesting that engagement in AI ethics training is essential for building employee knowledge and embedding responsible practices institutionally. Organizational AI ethics commitment correlated moderately with both performance (r = 0.624) and maturity (r = 0.559), indicating that while leadership commitment is valuable, it alone does not guarantee outcomes unless combined with training and competencies. Commitment also correlated modestly with training engagement (r = 0.497) and competencies (r = 0.58), highlighting that top-down values must be reinforced through concrete HR practices.

**Table 4:** The Fornell-Larcker criterion used for discriminant validity

Index	OAEC	RAIP	RAIC	AETE	RAIM
Organizational AI ethics commitment (OAEC)	**0.841**				
Responsible AI performance (RAIP)	0.624	**0.872**			
Responsible AI competencies (RAIC)	0.581	0.658	**0.854**		
AI Ethics training engagement (AETE)	0.497	0.536	0.609	**0.833**	
Responsible AI maturity (RAIM)	0.559	0.672	0.703	0.571	**0.864**

Note: Diagonal values in bold represent the square root of the average variance extracted (AVE) for each construct, while the off-diagonal elements represent the inter-construct correlations. According to the Fornell–Larcker criterion, discriminant validity is established when the square root of AVE for each construct exceeds its correlations with other constructs.

The heterotrait-monotrait ratios (Table 5) further supported discriminant validity, with all constructs falling below the 0.85 threshold. Strong associations were observed between training engagement and competencies (r = 0.596) and between maturity and performance (r = 0.779), confirming the hypothesized pathways. Together, these results confirm the reliability, discriminant validity, and structural integrity of the measurement model.

**Table 5:** The heterotrait-monotrait ratio used for discriminant validity

Index	OAEC	RAIP	RAIC	AETE	RAIM
Organizational AI ethics commitment (OAEC)	–				
Responsible AI performance (RAIP)	0.791	–			
Responsible AI competencies (RAIC)	0.745	0.697	–		
AI Ethics training engagement (AETE)	0.498	0.478	0.596	–	
Responsible AI maturity (RAIM)	0.803	0.779	0.811	0.608	–

Note: All heterotrait-monotrait ratio values are <0.85 (conservative criterion), indicating discriminant validity.

### Examination of the structural model

**Figure 2:**
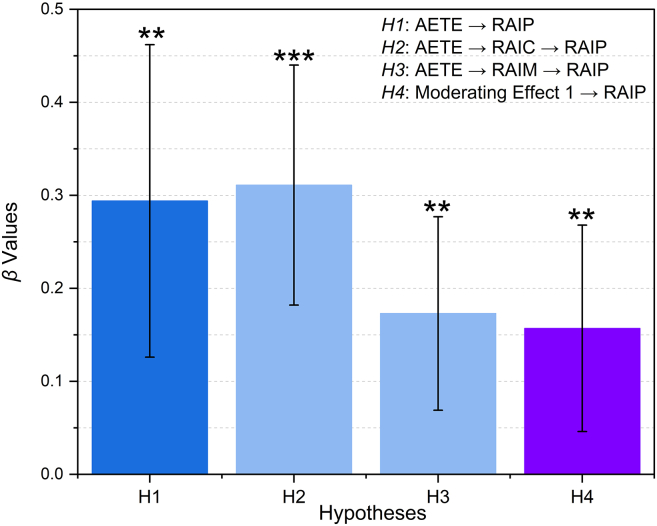
Structural model estimates for hypotheses testing Bars represent standardized path coefficients (β) estimated in SmartPLS 4.0. Error bars indicate the standard deviation of β across 5,000 bootstrap resamples (two-tailed inference). Significance levels are based on bootstrapped *p*-values: ∗∗*p* < 0.05; ∗∗∗*p* < 0.01. Abbreviations: AI ethics training engagement (AETE), responsible AI performance (RAIP), responsible AI competencies (RAIC), responsible AI maturity (RAIM).

The structural model was assessed using SmartPLS version 4.0, with path significance determined using 5000 bootstrap resamples. Overall, the model demonstrated strong explanatory and predictive capability. It confirmed the hypothesized relationships between AI ethics training, responsible AI competencies, responsible AI maturity, organizational commitment, and responsible AI performance. AI ethics training engagement had a significant positive effect on responsible AI performance (β = 0.294, *p* < 0.05). This result shows that employees who participate actively in ethics training are more capable of applying fairness, transparency, and accountability principles in real service contexts. The direct effect is illustrated in Figure 2. The mediation analyses confirmed that responsible AI competencies partially transmitted the influence of AI ethics training on performance (β = 0.311, *p* < 0.01). Employees who developed stronger competencies were better equipped to interpret algorithmic outputs critically and apply ethical reasoning in operational decision-making. Responsible AI maturity also exhibited a significant mediating effect (β = 0.173, *p* < 0.05). This finding highlights that training contributes not only to individual learning but also to the institutionalization of ethical procedures, oversight systems, and governance protocols. Training therefore strengthens organizational capacity to integrate ethical principles systematically into AI-supported processes. Organizational commitment to AI ethics positively moderated the association between maturity and performance (β = 0.157, *p* < 0.05). Firms with strong leadership commitment achieved higher performance at equivalent maturity levels than firms with weaker commitments. This demonstrates that governance systems are most effective when aligned with leadership values, resource allocation, and strategic communication. The model displayed considerable explanatory power, with R^2^ values of 0.493 for responsible AI competencies, 0.517 for responsible AI maturity, and 0.694 for responsible AI performance. Predictive relevance was confirmed by Q-square values above zero, indicating that the model has strong predictive accuracy. These findings demonstrate that training, competencies, maturity, and leadership commitment function collectively as an integrated system that drives responsible AI outcomes in service organizations.

## Discussion

This study examined how AI ethics training, responsible AI competencies, responsible AI maturity, and organizational commitment to ethical AI jointly influence responsible AI performance in hospitality and tourism enterprises. The findings highlight the interdependence of individual, organizational, and leadership factors in shaping ethical outcomes in AI-supported service environments. The review integrates these empirical results with resource-based theory, institutional perspectives, and HRM frameworks. The direct effect of AI ethics training on responsible AI performance confirmed that structured learning activities enhance employees’ ability to apply principles of fairness, transparency, and accountability. Training equips employees with the knowledge required to recognize algorithmic risks, evaluate outputs, and exercise appropriate human oversight.^3^^,^^9^ This finding reinforces the view that training is a practical mechanism for translating ethical aspirations into day-to-day decision practices.^1^^,^^23^ From a resource-based perspective, AI ethics training serves as an enabling resource because it builds knowledge and awareness that are valuable and not readily transferable across organizations. At the same time, institutional theory suggests that training responds to the broader societal expectations surrounding transparency and fairness. Training therefore serves dual roles as both a human capital investment and a response to legitimacy pressures.^18^^,^^40^

The mediation analysis offered information about how training influences performance. Responsible AI competencies strengthened the pathway from training to performance. This study shows that employees who internalize ethical reasoning and acquire skills in bias identification and interpretability are better positioned to make responsible decisions in service settings. Study results support earlier research linking these competencies to technology acceptance, ethical judgment, and organizational learning.^9^^,^^27^ Responsible AI maturity also mediated the relationship between training and performance. Research indicates that training contributes to the institutionalization of governance structures such as audit procedures, risk assessment practices, and communication routines. These processes create a consistent framework that supports employees in applying ethical principles across different service operations.^37^^,^^41^ Together, the two mediators highlight that responsible AI performance arises from both internalized competencies and organization-wide systems. This dual mechanism aligns with the adaptive management framework in HRM,^6^^,^^11^ where training enhances ability, organizational systems provide opportunity, and motivation is reinforced through leadership commitment.

The moderation effect confirmed that organizational commitment to AI ethics strengthens the influence of maturity on performance. Leadership commitment involves strategic communication, policy endorsement, and resource allocation, all of which help employees interpret governance practices as organizational priorities rather than technical requirements.^11^^,^^30^ Although the magnitude of the moderation was statistically modest, it is practically meaningful. Organizations with similar levels of maturity performed differently depending on leadership commitment.^21^^,^^42^ This confirms that governance systems function most effectively when supported by values, communication, and incentives at the managerial level.^43^^,^^44^^,^^45^ Leadership thus creates the cultural context that enables governance structures to influence performance.^37^^,^^46^

### Theoretical contributions

This study advances the theoretical understanding of responsible AI in several meaningful ways. First, it reframes AI ethics training as a strategic mechanism that supports responsible digital transformation. Prior research often treated training as a compliance activity.^15^ However, the findings of this study show that training contributes to capability development, institutional readiness, and AI ethics in service ecosystems. By demonstrating that training influences both individual competencies and organizational maturity, the study clarifies the strategic position of training in building responsible AI capacity. Second, the study differentiates responsible AI competencies and responsible AI maturity as distinct but complementary constructs. Competence operates at the individual level and reflects employees’ cognitive, ethical, and interpretive abilities.^2^^,^^19^ Maturity operates at the organizational level and reflects structured systems that include audits, oversight processes, and ethical guidelines. This separation responds to literature concerns about conceptual overlap and provides a clear framework for future research. Together, these constructs create a dual mechanism that explains how training supports responsible AI performance. Third, the study integrates resource-based and institutional perspectives to explain ethical outcomes at AI-supported workplaces. Training and competencies represent human capital resources that improve decision-making quality.^23^^,^^40^ Maturity and leadership commitment reflect institutional capabilities that strengthen organizational accountability and legitimacy. Combining these theoretical perspectives, the study shows how organizations align internal capabilities with external expectations for transparency, fairness, and ethical governance. Fourth, the study advances the understanding of leadership’s role in AI ethics by examining organizational commitment to AI ethics as a moderating variable. Leadership commitment strengthens the influence of maturity on performance. This indicates that formal governance structures are most effective when supported by clear values and strategic guidance.^47^ This finding enhances theoretical explanations of how culture and governance interact in responsible AI adoption.

### Practical contributions

The findings offered several practical implications for managers, human resource practitioners, and policymakers in the service industries. For managers,^48^^,^^49^ the results indicated that ethics training is an essential investment in preparing employees to use AI responsibly. Training could be designed not as a single session but as a continuous learning process that includes scenario-based activities, reflective discussions, and updates on emerging risks.^27^^,^^28^ Organizations may also ensure that the training is tailored to the responsibilities of employees who rely on AI tools in recruitment, customer service, and operational decision-making. For organizations, the results highlight the importance of building a responsible AI maturity. Ethics training alone is not sufficient. Firms may develop structured governance systems that include algorithmic auditing, risk assessment procedures, clear communication channels, and transparent oversight mechanisms. These systems help ensure that employees who acquire ethical competencies through training can apply them consistently in practice.^29^^,^^30^ Leadership commitment is central to responsible AI implementation. When executives communicate ethical priorities, allocate resources for oversight, and model responsible technology use, they create a culture that reinforces responsible AI behavior. Leadership engagement signals organizational expectations and increases confidence among employees who are expected to make ethical decisions when interpreting AI outputs. For policymakers, the research emphasizes the value of national and regional initiatives that support workforce development in ethical AI. Governments can strengthen responsible AI use across industries by funding training programs, establishing certification systems, and encouraging organizations to adopt transparent governance frameworks. Regulatory bodies can also encourage organizations to report on responsible AI practices as part of corporate sustainability disclosure requirements.

### Limitations of the study

This study has several limitations which provide opportunities for future research. The first limitation is the cross-sectional design. Although the study showed strong associations between training, competencies, maturity, leadership commitment, and performance, causal inferences cannot be made. Future research may use longitudinal or experimental designs to capture changes over time and clarify developmental processes that lead to responsible AI capability. The second limitation concerns self-reported measurements. Although procedural steps were taken to reduce common method bias, including separated measurement points, self-perceptions can still influence responses. Future studies may incorporate objective indicators such as third-party audit results, algorithmic fairness metrics, or supervisor evaluations of employee behavior. The third limitation relates to the regional context. The study was conducted in Southwest China, which has unique institutional characteristics and policy environments that support rapid digital transformation. Although this context provides rich insights, the results may not generalize to regions with different regulatory frameworks or cultural expectations. Comparative studies across countries and sectors would help clarify the influence of institutional and cultural factors on responsible AI adoption. A fourth limitation is the potential influence of demographic moderators. Variables such as age, education, digital experience, and job role may influence how employees respond to training and how they apply responsible AI competencies. Future research may explore these moderators to understand how different groups integrate ethical AI practices into their daily work. Finally, responsible AI is an emerging domain. Although this study adapted validated instruments from sustainability and governance research, further refinement of measurement tools is needed to capture the unique characteristics of responsible AI. Future research needs to develop and validate context-specific scales that reflect AI governance complexity.^50^^,^^51^^,^^52^

## Resource availability

### Lead contact

Requests for further information and resources should be directed to and will be fulfilled by the lead contact, Muhammad Arif (arifgreenexpert@outlook.com).

### Materials availability

This study did not generate new unique reagents.

### Data and code availability

• Data reported in this paper will be shared by the lead contact upon request.
• This paper does not report original code.
• Any additional information required to reanalyze the data reported in this paper is available from the lead contact upon request.

## Acknowledgments

The authors sincerely thank all anonymous participants who voluntarily took part in this study and shared their time and insights. Their contributions were essential to the completion of this research. This research was funded by the 2024 Ministry of Education Humanities and Social Sciences Research Project for Western and Border Regions (no. 24XJC630001); Project for Enhancing the Capacity of Popular Science Services in Ecological Conservation and Sustainable Utilization of Landscape Resources in the Li River Basin (2025FN9653478); Humanities and Social Science Project (2023QGRW049); 2023 Guangxi Philosophy and Social Sciences Research Project (23FYJ058); Guangxi Philosophy and Social Sciences Research Project 2024 (24SHF007); and Research Projects of the Scientific Research Team of the School of Tourism Ecology and Environment at 10.13039/100032580Guilin Tourism University in China (no. STKY-2024001).

## Author contributions

Conceptualization, M.A.; methodology, M.A., L.Z., X.Z., K.L., Z.C., Y.A., and M.T.; investigation, M.A., L.Z., X.Z., K.L., Z.C., Y.A., and M.T.; writing—original draft, M.A.; writing—review and editing, M.A., L.Z., X.Z., K.L., Z.C., Y.A., and M.T.; funding acquisition, M.A.; resources, M.A.; supervision, M.A.

## Declaration of interests

The authors declare no competing interests.

## Declaration of generative AI and AI-assisted technologies in the writing process

During the preparation of this work, the author(s) used Wordtune and Grammarly in order to fix grammatical errors and enhance understanding of context and meaning. After using this tool or service, the author(s) reviewed and edited the content as needed and take(s) full responsibility for the content of the publication.

## STAR★Methods

### Key resources table

**Table:** 

REAGENT or RESOURCE	SOURCE	IDENTIFIER
**Softwareand algorithms**		

SmartPLS 4.x	Ringle, C. M., Wende, S., & Becker, J. M. (2022). SmartPLS 4. SmartPLS GmbH, Oststeinbek, Germany	https://smartpls.com
OriginPro 2025	OriginLab Corporation. OriginPro, Version 2025. Northampton, MA, USA	https://www.originlab.com/originpro

### Experimental model and study participant details

#### Human participants

This study involved human participants employed in hospitality and tourism organizations in Southwest China, including hotels, online travel agencies, tour operators, and eco-lodges. Participants were adult employees actively engaged in service operations where AI-enabled systems are present or under implementation. The final analytic sample consisted of 279 employees (*n* = 279), where *n* represents the number of individual survey respondents included in the structural model estimation. The overall response rate was 91.475%. Participants ranged in age from 18 to 60 years, with the largest proportion between 18–30 years (42.294%). Gender distribution was 52.330% female and 47.670% male. No additional demographic categories such as race or ethnicity were collected due to contextual and regulatory considerations within the study region. All participants voluntarily completed the survey. No personally identifiable information was collected. Ethical approval was obtained from the relevant Institutional Review Board, and the study complied with institutional and national research ethics guidelines. Informed consent was obtained electronically prior to participation.

### Method details

#### Study area

Southwest China was selected as the study area (20.54°N–34.19°N, 97.34°E–112.04°E) because it represents a region experiencing rapid digitalization, expanded tourism development, and strong policy support for AI integration into service systems. The region includes multiple UNESCO-designated tourism destinations,^38^ which have implemented advanced digital tools to manage visitor flows, environmental monitoring, and service delivery.^47^ These factors create an ecosystem where AI applications directly influence workforce management, service quality, and sustainability reporting. At the same time, the region’s tourism and hospitality enterprises face increasing public expectations regarding the ethical use of data, the fairness of algorithmic decision systems, and transparency in AI-supported processes. This unique combination of technological innovation and ethical sensitivity provides an analytically rich context for investigating responsible AI governance. Prior studies describe Southwest China as a key site for technology-enabled tourism innovation,^8^^,^^10^ making it a relevant setting for evaluating how training, employee competencies, and organizational maturity shape AI outcomes in practice.^9^^,^^12^

#### Sampling and data collection

The statistical unit of this study is individual employees. Although respondents were accessed through their employing organizations, the data analysis focused exclusively on employee-level responses. This approach is consistent with studies that examine training, competencies, and perceptions of governance systems in service organizations. To obtain a diverse and representative sample, a stratified random sampling strategy was used. Four strata were defined based on the official accreditation categories of the China Association of Travel Services. These included hotels, online travel agencies, tour operators, and eco lodges. From each stratum, organizations were randomly selected using publicly available accreditation lists. After organizations agreed to participate, employees were randomly invited to complete the survey. This procedure ensured proportional representation across service sectors and reduced the likelihood of organizational-level selection bias. Sample adequacy was evaluated using G∗Power 3.1.9.2,^53^ which confirmed that at least 100 responses were required given medium effect sizes (f^2^ = 0.15), α = 0.05,^54^ and power = 0.95. PLS-SEM heuristics also recommend at least 10 times the maximum number of paths leading to a construct, which in this case required a minimum of 80 participants.^55^ The final sample consisted of 279 employees (out of 305, with a 91.475% response rate) from multiple organizations in the region. Respondents varied in age, education, job role, and experience, reflecting the demographic diversity of the local service workforce. Data were collected at two separate time points (April–June and November–December 2024) to reduce the common method variance. This method was to ensure that employees had sufficient time to reflect on their training experience and organizational practices. Although this design does not constitute a longitudinal study, it reduces the immediate perceptual bias that commonly occurs in single-time-point surveys. The survey was distributed both online and through workplace coordination, depending on the participating organizations' preferences. Online questionnaires were distributed using WeChat, which is a widely used communication platform in China. This method increased response rates but also introduced potential self-selection biases. This limitation has been described in the discussion section. Confidentiality was strictly maintained. Respondents were informed that participation was voluntary, responses were anonymous, and no identifying information would be linked to any individuals or organizations. All procedures were approved by the Institutional Review Board and adhered to ethical guidelines for research involving human participants.

#### Measurement instruments

All constructs in this study were measured using established scales, which were adapted to the context of responsible AI (Table 2). Each construct was measured on a seven-point Likert scale ranging from 1 (“strongly disagree”) to 7 (“strongly agree”), ensuring comparability with prior empirical studies. The adaptation process followed recommended procedures which included item review, contextual modification, back translation, and pilot testing to ensure linguistic clarity and cultural appropriateness. AI ethics training was measured using items that captured employees’ exposure to structured learning activities^56^^,^^57^ focused on algorithmic risks, fairness considerations, responsible data use, and ethical decision-making.^23^ Responsible AI competencies were measured using items that reflected employees’ ability^41^^,^^58^ to identify bias, interpret algorithmic output, understand transparency concerns, and apply ethical reasoning in practice.^27^ Responsible AI maturity was assessed using items adapted from governance and sustainability maturity models^59^ that include oversight structures, audit procedures, risk assessment systems, and institutional communication practices.^7^^,^^28^ Although some original instruments were developed in the context of environmental sustainability and HRM, their structural components, such as compliance systems and ethical oversight, are conceptually relevant to responsible AI. Items were adapted from replacing environmental terminology with AI-related terminology. For example, references to environmental audits were modified to algorithmic audits. In addition, references to compliance with environmental standards were modified to compliance with ethical AI guidelines. Organizational commitment to AI ethics was measured using items reflecting leadership priorities,^60^ resource allocation practices, strategic communication, and policy emphasis.^29^^,^^30^ Responsible AI performance was measured using items that assessed fairness, transparency, accountability, and responsible use of AI applications within service operations.^57^^,^^61^ To ensure semantic and cultural equivalence, the entire instrument underwent a back-translated process and a pilot test involving^30^ employees from the region. Feedback from the pilot tests was used to refine wording, improve clarity, and ensure that the items were understood as intended.

### Quantification and statistical analysis

The data were analyzed using SmartPLS version 4.0,^62^ which facilitated the robust examination of both measurements and structural models within the framework of PLS-SEM. The analyses followed a multi-stage process. First, descriptive statistics were employed to examine respondents’ demographic profiles, including gender, marital status, age, education level, and working experience. These profiles reflect their degree of exposure to AI-enabled technologies. Nonparametric tests, such as the Kruskal–Wallis test, were applied to evaluate subgroup differences. Results indicated significant variations in attitudes toward AI ethics training between younger and older employees. Second, the reliability and validity of the measurement model were assessed. Cronbach’s alpha (α) and composite reliability values exceeded the recommended threshold of 0.70, demonstrating strong internal consistency, while average variance extracted (AVE) values surpassed the 0.50 cutoff, confirming convergent validity. Discriminant validity was evaluated using the Fornell–Larcker criterion and the heterotrait–monotrait ratio, both of which confirmed that the constructs were conceptually distinct from one another.^54^^,^^55^ Third, potential multicollinearity was addressed by calculating variance inflation factors (VIFs) for each indicator, with all values falling below the standard threshold of 5.0 and almost all below 3.0, thereby eliminating concerns about collinearity bias.^63^^,^^64^^,^^65^ Finally, the structural model was evaluated using a bootstrapping procedure with 5,000 resamples to estimate the significance of path coefficients, including the hypothesized direct, mediating, and moderating relationships.^66^ The explanatory power of the model was assessed through R^2^ values, which indicated substantial variance explained in the dependent variable, while predictive relevance was confirmed through Q^2^ statistics. To complement the statistical results, graphical outputs, including structural path diagrams, standardized coefficient plots, and bootstrapped confidence intervals, were generated using Origin Pro 2025. This comprehensive analytical strategy ensured methodological rigor and offered clear empirical evidence for the relationships between AI ethics training engagement, responsible AI competencies, organizational maturity, leadership commitment, and responsible AI performance.

## References

[1] Meleti E. (2025). Human sustainability training and job satisfaction in organizations. Sustain. Dev..

[2] Koteczki R., Csikor D., Balassa B.E. (2025). The role of generative AI in improving the sustainability and efficiency of HR recruitment process. Disc. Sustain.

[3] Hao F., Ng W., Aman A.M., Zhang C. (2025). Technology for sustainability: the impact of Avatar-led Green Training on OCBE and green creativity. Int. J. Contemp. Hosp. Manag..

[4] Luu T.T. (2025). Linking big data on environmental conservation and predictive analytics to market pioneering of hotel firms: can green open innovation intervene?. Int. J. Hosp. Manag..

[5] Rosa F.S., Lunkes R.J., Codesso M., Mendes A.C.A., Costa G.D. (2025). Effects of green innovation ecosystem coopetition, environmental management practices and digital innovation on carbon footprint reduction. Int. J. Contemp. Hosp. Manag..

[6] Cao Y., Yan B., Teng Y. (2023). The impact of green human resource management on hospitality employees’ quitting intention: A dual perspective study. J. Hosp. Tour. Manag..

[7] Onijigin T., Özgit H., Ilkhanizadeh S. (2023). The nexus between organisational identification and employees’ behavioural outcomes: evidence from ecotourism businesses. Sustainability.

[8] Ai Y., Tian M., Chen Z., Arif M. (2025). Hospitality and tourism trends drive spatial shifts in urban service accessibility. Sustain. Cities Soc..

[9] Han S., Zhang H., Li H., Xun Z. (2025). Digital transformation and carbon emission reduction: the moderating effect of external pressure and support. J. Clean. Prod..

[10] Deng F., Tian Q., Arif M. (2025). Assessing the shifts in spatiotemporal ecotourism accessibility driven by high-speed rail development in China. Habitat Int..

[11] Pham T.D.,F.,F.D.X., Jiaying L., Liu D. (2025). Behave green or not: conceptualising the environmentally responsible behaviour of Vietnamese generation Z tourists and its transition process. Curr. Issues Tour.

[12] Tao A., Wang C., Zhang S., Kuai P. (2024). Does enterprise digital transformation contribute to green innovation? Micro-level evidence from China. J. Environ. Manage..

[13] Zhang H., Wu J., Mei Y., Hong X. (2024). Exploring the relationship between digital transformation and green innovation: the mediating role of financing modes. J. Environ. Manage..

[14] Wei X., Su L., Du J. (2025). Every cloud has a silver lining: understanding how hotel employees’ counterproductive work behavior may shape their pro-environmental behavior. Tour. Manag..

[15] Alkire L., Bilgihan A., Bui M.M., Buoye A.J., Dogan S., Kim S. (2024). RAISE: leveraging responsible AI for service excellence. J. Serv. Manag..

[16] Arif M., Yu H. (2026). Digital crisis communication in hospitality and tourism: public and industry perspectives from China. J. Hosp. Mark. Manag..

[17] Li H., Xi J., Hsu C.H.C., Yu B.X.B., Zheng X.K. (2025). Generative artificial intelligence in tourism management: an integrative review and roadmap for future research. Tour. Manag..

[18] Kim M.J., Hall C.M., Chung N. (2024). The influence of AI and smart apps on tourist public transport use: applying mixed methods. Inf. Technol. Tourism.

[19] Ferreira R.M.F.D., Grilo A., Maia M. (2025). Piloting a maturity model for responsible artificial intelligence: a portuguese case study. J. Responsib. Technol..

[20] Ikram M., Abahmaoui F.Z. (2025). Toward green growth in Morocco: an integrated strategic decision-making framework for sustainable development. Sustain. Dev..

[21] Rubel M.R.B., Kee D.M.H., Rimi N.N. (2025). Unpacking the eco-friendly path: exploring organizational green initiatives, green perceived organizational support and employee green behavior. Int. J. Contemp. Hosp. Manag..

[22] Lee S.U., Perera H., Liu Y., Xia B., Lu Q., Zhu L., Cairns J., Nottage M. (2025). Integrating ESG and AI: a comprehensive responsible AI assessment framework. AI Ethics.

[23] Mahade A., Elmahi A., Alomari K.M., Abdalla A.A. (2025). Leveraging AI-driven insights to enhance sustainable human resource management performance: moderated mediation model: evidence from UAE higher education. Disc. Sustain.

[24] Juo W.J., Wang C.-H. (2025). Clarifying the effect of green demarketing on sustainable performance in the service industry: does green learning matter?. Int. J. Hosp. Manag..

[25] Başer M.Y., Kozak M., Büyükbeşe T. (2025). Integration of emerging technologies in tourism and hospitality curriculum: an international perspective. J. Hosp. Leis. Sport Tour. Educ..

[26] Arif M., Amin H., Zarif N., Xiangyue L., Yukun C. (2024). Ecological-economic assessment of forest land degradation neutrality in the Indus River Basin of Pakistan. Environ. Dev. Sustain..

[27] Mollah M.A., Rana M., Amin M.B., Sony M.M.A.A.M., Rahaman M.A., Fenyves V. (2024). Examining the role of AI-augmented HRM for sustainable performance: key determinants for digital culture and organizational strategy. Sustainability.

[28] Nawaz N., Arunachalam H., Pathi B.K., Gajenderan V. (2024). The adoption of artificial intelligence in human resources management practices. Int. J. Inf. Manag. Data Insights.

[29] Papagiannidis E., Mikalef P., Conboy K. (2025). Responsible artificial intelligence governance: a review and research framework. J. Strateg. Inf. Syst..

[30] Singh R., Joshi A., Dissanayake H., Nainanayake D., Kumar V. (2025). Harnessing Artificial Intelligence and Human Resource Management for Circular Economy and Sustainability: A Conceptual Integration. Sustainability.

[31] Campos C., Dias A.C., Quinteiro P., Gutiérrez D., Villanueva-Rey P., Gallego M., Oliveira S., Laso J., Albertí J., Bala A. (2024). Assessing the environmental impacts of three different types of accommodations in Portugal and Spain by using an LCA approach. Sci. Total Environ..

[32] Dalla Vecchia A., Migliorini S., Quintarelli E., Gambini M., Belussi A. (2024). Promoting sustainable tourism by recommending sequences of attractions with deep reinforcement learning. Inf. Technol. Tourism.

[33] Joyce E. (2024). Rewilding tourism in the news: power/knowledge and the Irish and UK news media discourses. Ann. Tour. Res..

[34] Lin M., Zhonghe Z., Arif M. (2025). The intersection of digital transformation and environmental responsibility in traditional manufacturing enterprises amid new productive forces. J. Clean. Prod..

[35] Wang S., Tian Q., Chen X., Zhang Q., Deng F., Arif M. (2024). Study of the evolving relationship between tourism development and cultural heritage landmarks in the eight Chengyang scenic villages in China. Ecol. Indic..

[36] Febrianda R., Ariyani L., Hermawati W., Aminullah E., Dinaseviani A. (2025). Model of entrepreneurship and digital technology in responding to green and non-green innovation: case study of Batik SMEs in Indonesia. Sustain. Dev..

[37] Guo X., Xu J. (2024). Can urban digital intelligence transformation promote corporate green innovation? Evidence from China. J. Environ. Manage..

[38] Wang S., Liping Y., Arif M. (2025). Evolutionary analysis of ecological-production-living space-carrying capacity in tourism-centric traditional villages in Guangxi, China. J. Environ. Manage..

[39] Wang L., Jiang Z., Hu C., Zhao J., Zhu Z., Chen X., Wang Z., Liu T., He G., Yin Y., Lee D.-H. (2025). Comparing AI and human decision-making mechanisms in daily collaborative experiments. iScience.

[40] Lee K.Y., Sobhaeerooy R., Sheehan L. (2025). Navigating the digital transformation of ocean tourism industries: insights from the literature and industry experts. Inf. Technol. Tourism.

[41] Bavik A., Kuo C.-F. (2025). Systematic review of meta-analytic research in tourism and meta-analytic guidelines for social science. Serv. Ind. J..

[42] Lin M., Zhonghe Z., Wei F., Arif M. (2025). Trust and communication in supervisor-subordinate relationships in technology-intensive business sectors in China. Humanit. Soc. Sci. Commun..

[43] Dubey A., Yang Z., Hattab G. (2024). A nested model for AI design and validation. iScience.

[44] Hamed A.A., Zachara-Szymanska M., Wu X. (2024). Safeguarding authenticity for mitigating the harms of generative AI: issues, research agenda, and policies for detection, fact-checking, and ethical AI. iScience.

[45] Longin L., Bahrami B., Deroy O. (2023). Intelligence brings responsibility - Even smart AI assistants are held responsible. iScience.

[46] Tuo Y., Wu J., Zhao J., Si X. (2025). Artificial intelligence in tourism: insights and future research agenda. Tour. Rev..

[47] Li Q., Wang X., Chen Z., Arif M. (2024). Assessing the conjunction of environmental sustainability and tourism development along Chinese waterways. Ecol. Indic..

[48] Do T.K. (2025). Generative AI in service research: promise or peril?. Serv. Ind. J..

[49] Ivanov S. (2025). Responsible use of AI in social science research. Serv. Ind. J..

[50] Mussi M., Metelli A.M., Restelli M., Losapio G., Bessa R.J., Boos D., Borst C., Leto G., Castagna A., Chavarriaga R. (2025). Human-AI interaction in safety-critical network infrastructures. iScience.

[51] Ramchurn S.D., Stein S., Jennings N.R. (2021). Trustworthy human-AI partnerships. iScience.

[52] Yang G., Xiao Q., Zhang Z., Yu Z., Wang X., Lu Q. (2025). Exploring AI in metasurface structures with forward and inverse design. iScience.

[53] Shieh G. (2021). Probing categorical moderation under variance heterogeneity. Psychol. Methods.

[54] Lafit G., Revol J., Cloos L., Kuppens P., Ceulemans E. (2025). The effect of different construct operationalizations, study duration, and preprocessing choices on power-based sample size recommendations in intensive longitudinal research. Assessment.

[55] Giner-Sorolla R., Montoya A.K., Reifman A., Carpenter T., Lewis N.A., Aberson C.L., Bostyn D.H., Conrique B.G., Ng B.W., Schoemann A.M., Soderberg C. (2024). Power to detect what? Considerations for planning and evaluating sample size. Pers. Soc. Psychol. Rev..

[56] Teixeira A.A., Jabbour C.J.C., de Sousa Jabbour A.B.L., Latan H., de Oliveira J.H.C. (2016). Green training and green supply chain management: evidence from Brazilian firms. J. Clean. Prod..

[57] Masri H.A., Jaaron A.A.M. (2017). Assessing green human resources management practices in Palestinian manufacturing context: an empirical study. J. Clean. Prod..

[58] Cabral C., Chiappetta Jabbour C.J. (2020). Understanding the human side of green hospitality management. Int. J. Hosp. Manag..

[59] Garcés-Ayerbe C., Rivera-Torres P., Murillo-Luna J.L. (2012). Stakeholder pressure and environmental proactivity Moderating effect of competitive advantage expectations. Manag. Decis..

[60] Raineri N., Paillé P. (2016). Linking corporate policy and supervisory support with environmental citizenship behaviors: the role of employee environmental beliefs and commitment. J. Bus. Ethics.

[61] Elhoushy S., Elzek Y., Font X. (2025). Sustainable tourism certification: a systematic literature review and suggested ways forward. J. Sustain. Tour..

[62] Li X., Law R. (2020). Network analysis of big data research in tourism. Tour. Manag. Perspect..

[63] Nguyen Thi Ha P., Tran Van H., Le Thai P., Trinh Thi Phuong T., Tran T. (2024). Partial Least Squares Structural Equation Modeling (PLS-SEM) in higher education research: an evidence from using Technology Acceptance Model (TAM) and Innovation Resistance Theory (IRT). New Rev. Inf. Netw..

[64] Guenther P., Guenther M., Ringle C.M., Zaefarian G., Cartwright S. (2023). Improving PLS-SEM use for business marketing research. Ind. Mark. Manag..

[65] Shela V., Ramayah T., Aravindan K.L., Ahmad N.H., Alzahrani A.I. (2023). Run! This road has no ending! A systematic review of PLS-SEM application in strategic management research among developing nations. Heliyon.

[66] Samara D., Magnisalis I., Peristeras V. (2020). Artificial intelligence and big data in tourism: a systematic literature review. J. Hosp. Tour. Technol..

